# Hemihydranencephaly: living with half brain dysfunction

**DOI:** 10.1186/1824-7288-39-3

**Published:** 2013-01-16

**Authors:** Piero Pavone, Francesco Nigro, Raffaele Falsaperla, Filippo Greco, Martino Ruggieri, Renata Rizzo, Andrea D Praticò, Lorenzo Pavone

**Affiliations:** 1 Unit of Pediatrics and Pediatric Emergency, Azienda Ospedaliera Universitaria Vittorio-Emanuele-Policlinico, Via Plebiscito 767, 95123, Catania, Italy; 2 Unit of Neonatology, Hospital Garibaldi, Catania, Italy; 3 Department of Pediatrics, University of Catania, Catania, Italy; 4 Department of Formative Processes, University of Catania, Catania, Italy; 5 Chair of Child Neurospychiatry, University of Catania, Catania, Italy

**Keywords:** Unilateral hydranencephaly, Congenital anomaly, Brain malformation, Carotid artery anomaly

## Abstract

Hemi-hydranencephaly is a very rare condition characterized by complete or almost near-complete unilateral absence of the cortical cortex, which is filled by a sac of cerebrospinal fluid. Prenatal vascular disruption with occlusion of the carotid artery territories ipsilateral to the damaged brain is the presumed pathogenesis.

We have selected nine cases that fit the clinical and pathologic characteristics of hemi-hydranencephaly, demonstrating that destruction of one hemisphere may be not always associated with severe neurologic impairment and may allow an almost normal life. This disorder is an example of a possible prenatal re-organization in which the right and left cerebral hemispheres present functional potentiality to make up the damaged brain.

The cases reported in the literature are discussed, including a patient previously reported and followed-up for 10 years. A review of the cases is performed with an evaluation of the most important aspect of this rare and mysterious disorder.

## Definition

Hemi-hydranencephaly (HHE) is a rare brain anomaly characterized by complete or near-complete unilateral absence of the cerebral cortex. The affected hemisphere is filled with cerebrospinal fluid (CSF) into membranous sacs, which also contains minimal residual neurons, glial tissue, and some blood vessels [1]. Usually, the meninges, basal ganglia, pons, medulla, cerebellum, and falx are not involved [1,2]. Unlike hydranencephaly, a disorder in which both cerebral hemispheres are completely or almost completely absent, the unilateral form may have a relatively good clinical outcome in many cases.

## Historical background

We are not aware of patients affected by HHE before the case reported by Warkany [3] in his fundamental book entitled “Congenital Malformations: Notes and Comments,” in which a photo given to Dr. N. Hayden (Seattle, WA, USA) of unilateral left cerebral involvement in a 3-year-old girl is demonstrated by transillumination. Prior to this report in 1959, Muir [4] reported the pathologic features of a deceased child 4 years of age who was diagnosed with bronchopneumonia. At the time of autopsy, the child had a slightly enlarged right cerebral hemisphere, a dilated lateral ventricle, and absence of the left frontal, temporal, and parietal lobes. The cerebellum, cerebral peduncles, and choroid plexus were not involved. The circle of Willis was normally structured with small vessels on the left and the middle cerebral vessels coursing laterally as far as the edge of the nerve tissue sheet. In our opinion, the pathologic description of this case raises some doubt about the diagnosis of HHE and will not be included in this review. Subsequently, a case of HHE was described by Moser and Seljeskog [5] in a 2-day-old newborn with a nearly complete absence of the left cerebral hemisphere and preservation of a very small margin of the medial occipital fold. The basal ganglia and thalamic structures were normal. In this patient, a combined pneumoencephalogram-ventriculogram showed a free communication between the right ventricle and left hemicranial fluid compartments. Two others patients with HHE were reported by Suzuki et al. [6] and Ohtsuka [7], but the clinical and neuroradiologic features, like the patient described by Muir [4], were consistent with a possible, but not definite case of HHE, and are thus not included in this review. In fact, the authors reported the differential diagnosis with hydrocephalus was not feasible for the former case and the clinical signs were complex, including the association with holoprosencephaly and other multiple congenital malformations, in the latter case. The first case report of HHE with a favorable outcome was described by Van Doornick and Hennekam [8] in a 5-year-old patient. In the last 10 years, 6 new cases of HHE have been described by Greco et al. [9], Ulmer (2; in a 36-year-old who presented with episodes of headaches), Altschuler et al. [10], Balpande et al. [11], Hassaneim et al. [12], and Dias et al. [13].

## Epidemiology

HHE is a very rare disorder. HHE is much rarer than hydranencephaly, which has been reported with a prevalence of 0.2% at autopsy [14]. The references for this review were obtained from PubMed using the search term, hemi-hydranencephaly. Among the cases reported, we have selected nine cases which meet all the criteria for typical HHE; specifically, unilateral involvement of a cerebral hemisphere filled by a sac containing cerebrospinal fluid (CSF).

## Pathogenesis: HHE as a prenatal vascular disruption

HHE is thought to be due to the occlusion of a unilateral carotid artery causing ipsilateral absence of a hemisphere that will be filled by CSF. The meninges, basal ganglia, pons, medulla, cerebellum, and falx, all of which are supplied by the vertebrobasilar system, are not involved. The anomalous event should have occurred after neural migration and before synaptogenesis [15]. During this period, the fetal cerebral hemisphere shows an important transformation with wide proliferation, which will cover the diencephalon, mesencephalon, and entire brainstem. In patients with HHE, the affected cerebral hemisphere is initially formed, but subsequently presumed to be destroyed by a severe encephaloclastic process. The reason why the disorder affects only one carotid artery and the connected hemisphere is not known. In fact, the developing brain should be able to compensate for vessel occlusions via the circle of Willis and leptomeningeal collateral vessels.

In cases of hydranencephaly, many predisposing factors have been reported [3,9]. In contrast to hydranencephaly, no pathologic events were reported in the patients affected by HHE which we reviewed, with the exception of the patient reported by Hassaneim et al. [12], who had an anomalous level of protein S (30% [normal value, 70%-123%]) and antithrombin III (135% [normal value, 80%-120%]), and Doornik and Hennekam [8], the mother of whom had a severe influenza-like episode during the fourth month of pregnancy.

Unilateral induced hydranencephaly has been demonstrated in monkeys after *in utero* ipsilateral ligation of the carotid artery and jugular veins [16] and newborn puppies by the injection of heated paraffin into the carotid artery [17]. Recently, somatic mutations in specific genes pathway PI3K-Akt3-mTOR have been implicated in the pathogenesis of neurodevelopmental disorders and specifically in megalencephaly syndrome. These genes pathway intervene in multiple cellular function and could play a role in the pathogenesis of the hemihydrancencephaly [18-20].

The formation of cavities resulting by a hypoxic-ischemia injury, as one of the possible causes of HHE, is a peculiar response to the developing fetal brain where the macrophage response to cell death is present and the neuroglial response is still developing [21]. In patients with HHE, the hemisphere is initially formed, but later destroyed by the encephaloclastic process with disappearance of the cerebral structures, including the ventricular wards [22].

### Preservation of cognitive performance in patients with HHE

Why is such an impressive anatomic anomaly infrequently associated with severe mental retardation and/or language delay? First, independently of which hemisphere is affected, there are no differences with respect to clinical signs and outcomes.

Most of the reported cases of HHE in the literature, including our patient, show that progressive learning is possible across many cognitive domains in patients with a single hemisphere. There have been many reports of cognitive performance outcomes in children who have undergone hemispherectomies for severe and intractable seizures [23]. Pulsifer et al. [24] reported cognitive outcomes in 71 children who underwent hemispherectomies and found little changes in cognitive performance pre- and post-surgery. The series had a mean age at surgery of 7.2 years. There is a fundamental difference between a patient with HHE and a patient who has undergone a hemispherectomy; specifically, the former patient has no possibility of interhemispheric connection after birth, and for a varying period of time *in utero*, thus giving a clear picture of the functional potential of a single cerebral hemisphere. The results obtained from the literature show that relative preservation of cognitive performance suggests that a single cerebral cortical hemisphere connected to an apparently intact brainstem is sufficient for the development of higher cognitive function. According to the side affected, there is no outcome difference; a good or severe cognitive development may be possible whether or not the left or right hemisphere is involved. Good cognitive development has been reported [2,11], in which the left and right hemispheric lesions were involved, respectively. According to the quite good or good cognitive development seen in some of these patients, it is correct to think that cortical re-organization is possible when the damage occurs in a very early stage. How and when plasticity occurs is not known. As reported by Ulmer [2], activation of an already existing pathway, development of new connections, axonal migration, or sprouting may create the pattern for a stable and constructive re-organization, which results in a good outcome in patients affected by unilateral right or left hydranencephaly. There are reasons to think that in each side of the developing brain there exists a structural anatomic pathway with the potential to reach a partial or total absence of the initial function of the destroyed hemisphere. However, in addition to cases with good outcomes HHE may result in severe cognitive involvement and poor outcome [13]. The sample size of reported patients with HHE is small, but it is clear that patients with HHE are likely heterogeneous with respect to the mechanism and timing of injury, so it is clear that if we have cases in which no motor or cognitive impairment exists, there have been cases in which the pathologic event has caused severe damage.

## Clinical features of HHE

Table 1 summarizes the clinical data regarding the patients affected by HHE, including the patient reported by Greco et al. [9] who was seen by our group and reported in 2001 [1-13]. Currently, this child is 12 years old. The patient was periodically followed by one of us (P.P). He is attending Italian Junior high school and is performing well with a support teacher. He is able to construct phrases and engage in conversational speech and his speech is articulate. The neurologic examination shows a right hemiparesis, with right lower extremity hyperreflexia, convergent strabismus, and horizontal nystagmus. He walks without assistance with mild circumduction of the right lower extremity.

**Table 1:** Main characteristics of the reported cases of hemihydranencephaly

**Authors**	**Sex**	**Familiarity**	**Perinatal period**	**Age at diagnosis**	**Age of onset**	**Clinical signs**						**Radiological Findings**	**Affected Side**	**Annotations**
						**Motor Impairment Mental Retardat.**		**Seizures**	**Language**	**C.C.**	**Ocular Signs**			
Warkany, 1971	F	NR	NR	3½YY	NR	NR	NR	NR	NR	NR	NR	NO	L	Reported by Warkany
Moser 1981	NR	N	N	NR	7 MM	Right mild	NO	NR	NR	NR	NR	Absence left hemisphere	L	Shift towards middle; EEG: absence of left electrocerebral activity
Van Doormik, 1992	F	NO	N	5 YY	4 MM	Left Facial paralysis	IQ 69	NO	Delayed	NR	Strabismus,abducent paresis	Right side HHE	R	EEG: absence of electrocerebral activity on the right side. Reduced capacity of non verbal IQ and expressive language
												No middle right cerebral artery		
Greco et al., 2001	M	NO	Prematurity respiratory distress	4MM	Neonatal	Right	IQ 45	NO	Delayed	M	NR	Left side HHE	L	Hydrocephaly. EEG: absence of left electrocerebral activity
														IQ 45, low cognitive potential
Ulmer et al., 2005	M	NO	N	36 YY	Early Childhood	Right hemiparesis	N	One seizure 28 Years	N	NR	NR	Left side HHE	L	Mirror movements
												Left internal carotid artery		Motor acuity test : fine motor impairment
														Aachenen aphasia test: normal
Altshuler et al., 2005	M	NO	N	NR	3½ MM	Generalized hypertonia, more on the right	NR	NR	NR	NR	NR	Absence left hemisphere	L	-
Balpande M et al., 2009	M	NO	N	NR	13 YY	Left	N	NO	N	NR	-	Right side HHE	R	Diffuse atrophy, facial weakness
												Absence of internal right cerebral artery		
Hassanein et al., 2011	F	NR	NR	27 MM	NR	Left	Delayed mental milestones	NO	Delayed	≥ 3 C	Optic atrophy Nystagmus, bilateral blindness	Right side HHE	R	Diabetes insipidus.
												Right middle and anterior cerebral artery occlusion		Protein S deficiency
														Diffuse tensor MR: reduced
Dias LS et al., 2011	M	NO	N	21 YY	3 MM	Left	Severe	GTCS	Delayed 1,5 y	NR	Left asymmetric pupil	Right side HHE	R	-
								Status epilepticus				Internal carotid occlusion		

**Legend:***HHE*: hemihydranencephaly; *NR*: not reported; *NP*: not performed; *N*: normal; *YY*: years; *MM*: months; *M*: macrocephaly.

He has mild impairment of right hand function, but is able to perform functionally significant bimanual tasks. The visual-evoked potentials are normal in both eyes and the visual acuity is 20/30 bilaterally with correction. The visual fields show partial right homonymous hemianopia.

Based on the data of the cases of HHE reported in the literature, the family histories were negative and no similar cases were reported in the family members. Males were more frequently affected than females, with a male-to-female ratio of 5:3 (the gender was not reported in reference 5). The neonatal period was uneventful for all of the children, with the exception of the child reported by Greco et al. [9], who was born prematurely and also had neonatal respiratory distress. In most of the patients the clinical signs began in infancy, with age ranging from the neonatal period to infancy, with the exception of the case reported by Balpande [11], in whom the clinical signs were noted at 13 years of age. However, the disorder was diagnosed in most of cases at a later time due to the benign clinical course, and in two cases at 21 [13] and 36 years of age [2]. The left and right sides were affected in five and four patients, respectively.

The clinical signs presented by the affected patients were mainly neurologic (hemiparesis, language and/or cognitive impairment, and convulsive episodes). Ocular involvement was also reported. In one patient, HHE was associated with hydrocephaly [9] and in another patient a contralateral mass effect was diagnosed [5]; in both of these patients fluid drainage shunts were placed. Mirror movements were noted in the patient reported by Ulmer [2] in adulthood. Severe mental retardation was reported by Dias [13], light-to-moderate mental retardation by Van Doornik [8], Greco et al. [8], and Hassaneim [12], while patients with no mental retardation were reported by Moser [5], Ulmer [2], and Balpande et al. [11]. In two patients, cognitive outcomes were not reported. Convulsive episodes were reported in two patients, the patient described by Ulmer [2] had one generalized seizure, while the patient reported by Dias [12] had two episodes of generalized tonic-clonic seizures and an episode of status epilepticus.

Language delay has been reported in patients by Van Doornk [8], Greco et al. [9], Hasseneim et al. [12], and Dias [13]; in two patients the language was normal, and in three patients language skills were not reported.

Hemiparesis was reported on the right side in four patients [2,5,9,10], and on the left side in four patients [8,11-13].

### Diagnosis

**Figure 1:**
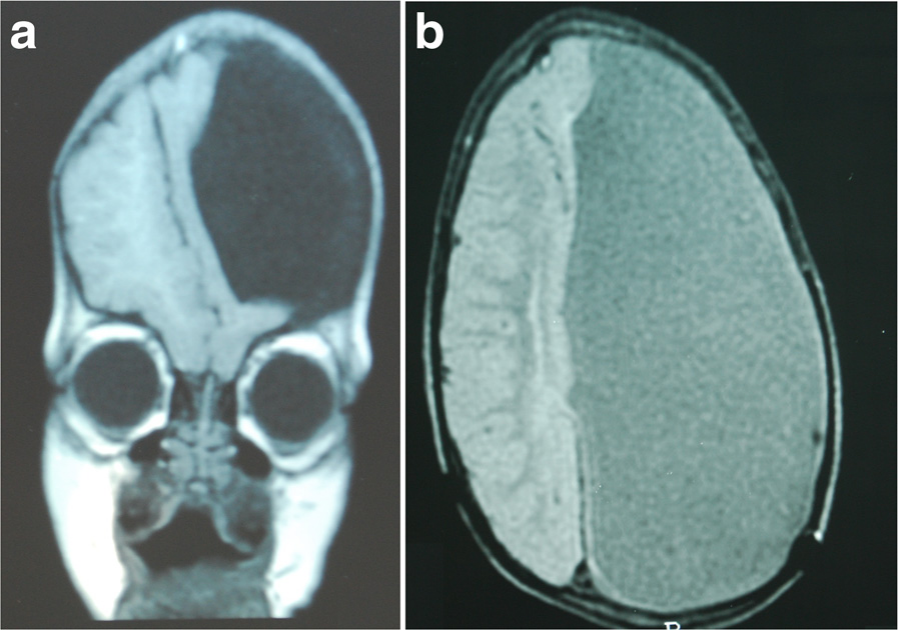
**a: MRI sagittal T1 scan demonstrating a left-sided hemihydranencephaly with replacement of the left cerebral cortex with cerebrospinal fluid-filled sacs at the age of 1 year. b**: MRI coronal T2 scan demonstrating left-sided hemihydranencephaly at the age of 2,5 year.

The diagnosis of HHE was established based on the neurologic clinical signs of motor impairment and/or mental delay, and with the use of neuroradiologic techniques (transillumination, CT scan, brain MRI, and cerebral angiography, see Figure 1). These findings facilitated the visualization of the absence of involvement of the unilateral hemisphere and angiography demonstrated the occlusion of the internal carotid arteries.

EEGs showed severe alterations in the side affected with asymmetry between the two hemispheres and absence of electric activity on the affected side.

Hassaneim et al. [12] performed diffusion tensor MR, and documented a reduced fractional anisotropy and higher diffusivity of the contralateral hemispheric tracts than a normal age-matched group. The authors concluded that there exists complete absence of the cortico-spinal and optic tracts in the side affected, combined with deficient contralateral tracts, and maintained that cortical re-organization may be impaired with early cerebral foetal events.

Neuropsychological testing reported by Van Doornik and Hennekam [8] in a 5-year-old girl gave the following results: Snijders-Oomen Non-verbal Intelligence Scale for young children (non-verbal IQ 69 [3 years, 9 months]); receptive language tested by the Dutch adaptation of the Peabody Picture Vocabulary Test gave a word age of 3.5 years, and expressive language evaluated by the Dutch adaptation of Christal’s Grammatical Analysis of language Disability spontaneous speech was equivalent to that of a child 2.5 years of age. In the case reported by Ulmer [2], the patient scored 64 of 85 possible points (75%) on the Wolf Motor function test due to a reduced speed and/or precision in almost all of the performed tasks and because of impaired fine motor control in three tasks; for language on the Aachenen aphasia test, the speech result was not affected. Our patient at the age of 12 years shows at WISC-III: verbal IQ 70; performance IQ 80; full-scale IQ was 72 (borderline area) [25-27].

### Co-morbidity

No other affections are associated with HHE. The patient reported by Hassaneim et al. [12] had diabetes insipidus, an unrelated disease. With respect to cerebral circumference, macrocephaly was reported by Greco et al. [9] and microcephaly was reported by Hassaneim et al. [12].

### Outcome

The disorder has a stable course. The patient who we followed for 10 years had good motor improvement due to motor rehabilitation and botulin treatment.

#### Treatment strategies

Treatment of this pathology is related to the clinical involvement shown by patients. Spasticity must be treated by physical rehabilitation therapy, with botulin, and when necessary, with orthopedic intervention. Logotherapy is useful for patients with language disturbances. Preventive anticonvulsant treatment is not recommended and ordered only in case of episodes of seizures. Neurosurgery may be requested when HHE is associated with hydrocephalic processes or a midline shift toward the normal hemisphere [5,9].

## Conclusions

On the base of the nine cases of HHE reported in the literature as typical for this affliction, we can summarize, as follows: 1) there is no difference in the clinical impairment and outcome regardless of which hemisphere is affected; 2) the right and left hemispheres are almost equally affected; 3) there is a higher prevalence of males affected compared to females; 4) hemilateral motor function is always involved to different extents; 5) cognitive and language functions are very often preserved with good or partial good performances; 6) heterogeneity in the clinical presentation and outcome probably depends on the mechanism and timing of the injury.

## Competing interest

The Authors declare that the present work has not been published previously, that it is not under consideration for publication elsewhere, that its publication is approved by all authors, and has been approved by the responsible authorities where the work was carried out. The Authors have no financial disclosures or competing interest to declare concerning this manuscript.

## Authors’ contributions

PP conceived the study, coordinated and wrote the article; FN, FG, ADP collected the data of the literature; MR, LP, RF, RR carried out the clinical diagnosis and followed up the patient. RR performed clinical and neuropsychological test evaluation. All authors read and approved the final manuscript.
